# Simultaneous Myocardial Infarctions in Dual Coronary Territories Due to a Single Culprit Lesion: A Diagnostic and Management Challenge

**DOI:** 10.7759/cureus.86658

**Published:** 2025-06-24

**Authors:** Nidhi Singh, Tushar Agarwal, Rishika Gupta, Amit Kumar, Vaibhav S Bhandary

**Affiliations:** 1 Department of Cardiology, Sarvodaya Hospital, Greater Noida West, IND; 2 Department of Radiology, Yatharth Hospital, Noida, IND; 3 Department of Pediatrics, King George's Medical University, Lucknow, IND; 4 Department of Radiology, Srinivas Institute of Medical Sciences and Research Center, Mangaluru, IND

**Keywords:** 12-lead ecg, coronary angiography, culprit lesion, percutaneous transluminal coronary angioplasty, st-elevation myocardial infarction (stemi)

## Abstract

Electrocardiographic (ECG) changes in myocardial infarction (MI) typically evolve predictably over time, allowing early identification of the ischemic territory and culprit vessel. Rarely, simultaneous ischemic changes in territories supplied by two different coronary arteries are observed, complicating diagnosis and urgent management. We present a rare case of acute chest pain with concurrent hyperacute T-waves in anterior leads, suggestive of left anterior descending (LAD) artery occlusion, and ST-segment elevations in inferior leads, suggestive of right coronary artery (RCA) occlusion. Coronary angiography revealed a chronic total occlusion of the RCA with retrograde filling from the LAD and an acute thrombotic lesion in the mid-LAD. Emergency percutaneous intervention in the LAD restored perfusion to both coronary beds, with rapid clinical and ECG recovery. This case highlights the importance of understanding ECG evolution in MI and emphasizes early revascularization strategies when facing diagnostic dilemmas.

## Introduction

Acute myocardial infarction (MI) remains a leading cause of morbidity and mortality worldwide. Rapid diagnosis and revascularization are critical to preserving myocardial function and improving outcomes [1]. The electrocardiogram (ECG) serves as a pivotal tool in identifying the location and timing of ischemia, with a well-described temporal sequence of changes, including hyperacute T waves, ST-segment elevation, Q-wave formation, and T-wave inversions [2,3]. Typically, ST-elevation patterns help localize the culprit artery; anterior leads (V1-V6) suggest left anterior descending (LAD) artery involvement, while inferior leads (II, III, and aVF) indicate right coronary artery (RCA) or left circumflex (LCX) artery pathology [4,5].

Simultaneous ST-segment elevations in two arterial territories are rare and pose diagnostic challenges. This phenomenon may occur due to concurrent occlusions or collateral dependence from a single artery supplying multiple territories [6]. We present an unusual case of simultaneous anterior and inferior lead involvement due to an acute LAD occlusion in a patient with prior RCA infarction and chronic occlusion, highlighting the importance of prompt interpretation and management.

## Case presentation

A 53-year-old man with a known history of hypertension and poorly controlled diabetes mellitus presented to the emergency department with sudden-onset, severe left-sided chest pain lasting for two hours. The pain was associated with profuse sweating and shortness of breath. He had a previous history of inferior wall myocardial infarction (IWMI) in 2009, treated with percutaneous transluminal coronary angioplasty to the RCA. Additionally, he had sustained a middle cerebral artery infarct one year before and was managed conservatively. He was on long-term antiplatelet and statin therapy but had defaulted on medications for the past month.

Upon initial examination, the patient was tachypneic, with a respiratory rate of 26 breaths/minute, a blood pressure of 102/68 mmHg, and a heart rate of 112 beats per minute. Fine bilateral basal crepitations were noted on auscultation (Killip Class 2), and his oxygen saturation was 92% on room air. Cardiovascular examination was otherwise unremarkable.

**Figure 1:**
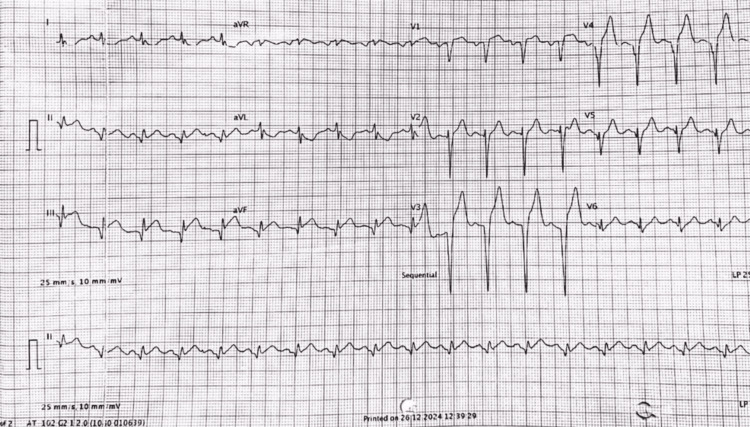
Initial 12-lead ECG showing fresh ST elevation in leads II, III and aVF with hyperacute T waves in leads V3-V4 ECG: electrocardiogram; aVR: augmented vector right; aVL: augmented vector left; aVF: augmented vector foot

**Figure 2:**
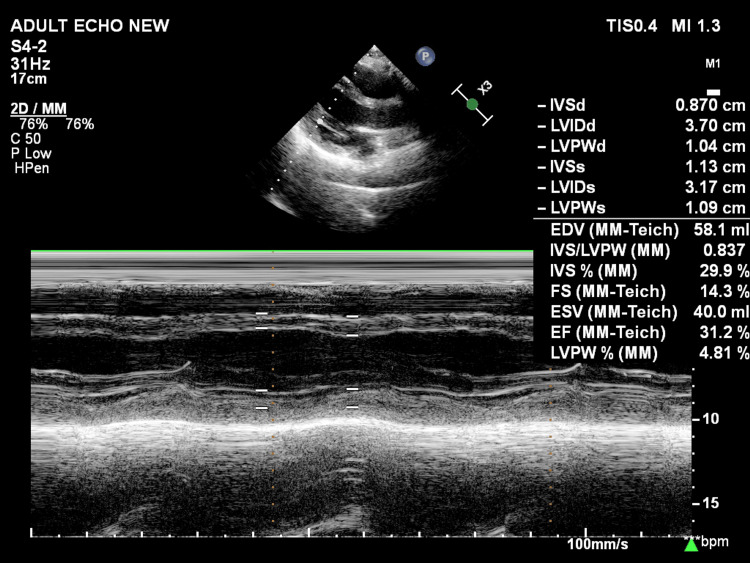
Initial two-dimensional echocardiography (M-mode) showing an EF of 30%-35% IVSd: interventricular septum thickness at end-diastole; LVIDd: left ventricular internal diastolic diameter; LVPWd: left ventricular posterior wall thickness in diastole; IVSd: interventricular septum thickness at end-systole; LVIDs: left ventricular internal systolic diameter; LVPWd: left ventricular posterior wall thickness in systole; EDV: end-diastolic volume; IVS: interventricular septum; LVPW: left ventricular posterior wall; FS: fractional shortening; ESV: end-systolic volume; EF: ejection fraction

The initial 12-lead ECG (Figure 1) demonstrated fresh ST-segment elevations in leads II, III, and aVF, with hyperacute T waves in V3-V4. This raised the possibility of acute occlusion involving both the RCA and LAD territories. Bedside two-dimensional echocardiography (Figure 2) revealed global left ventricular (LV) hypokinesia with a significantly reduced ejection fraction (EF) of 30%.

Emergency laboratory workup showed elevated cardiac troponins and mild leukocytosis. Renal parameters were normal. After administration of loading doses of aspirin and ticagrelor, as well as intravenous heparin, the patient underwent emergent coronary angiography. This revealed critical triple vessel disease: a 95% thrombotic occlusion in the mid-LAD, 100% chronic occlusion of the proximal LCX, and 100% chronic occlusion of the proximal RCA, with retrograde collateral filling from the LAD (Rentrop grade 3).

**Figure 3:**
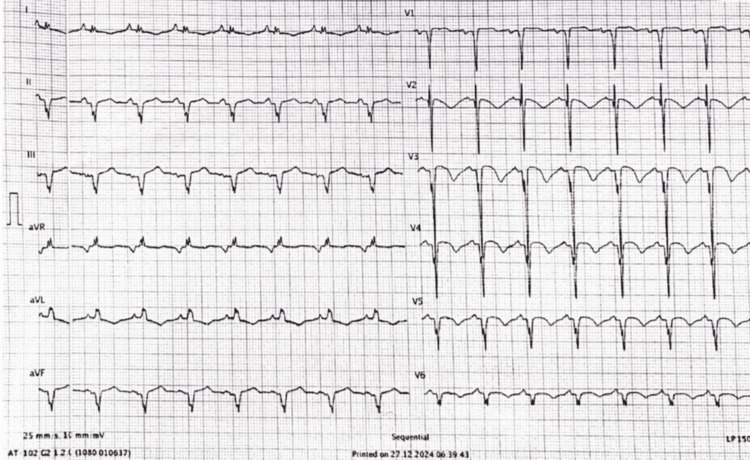
Repeat ECG 90 minutes after revascularization showing evolved T waves in anterior leads and ST segment returned to baseline in inferior leads ECG: electrocardiogram; aVR: augmented vector right; aVL: augmented vector left; aVF: augmented vector foot

Given the findings, the patient underwent primary percutaneous intervention to the mid-LAD lesion, utilizing a drug-eluting balloon. A drug-eluting stent was not implanted, keeping in view the need for coronary artery bypass grafting (CABG) after stabilization to achieve complete revascularization. Thrombolysis in myocardial infarction 3 flow was successfully restored. Postintervention ECG (Figure 3) showed resolution of ST-segment elevation in inferior leads and evolution of T wave inversions in anterior leads. The patient’s chest pain resolved, and hemodynamic status improved.

**Figure 4:**
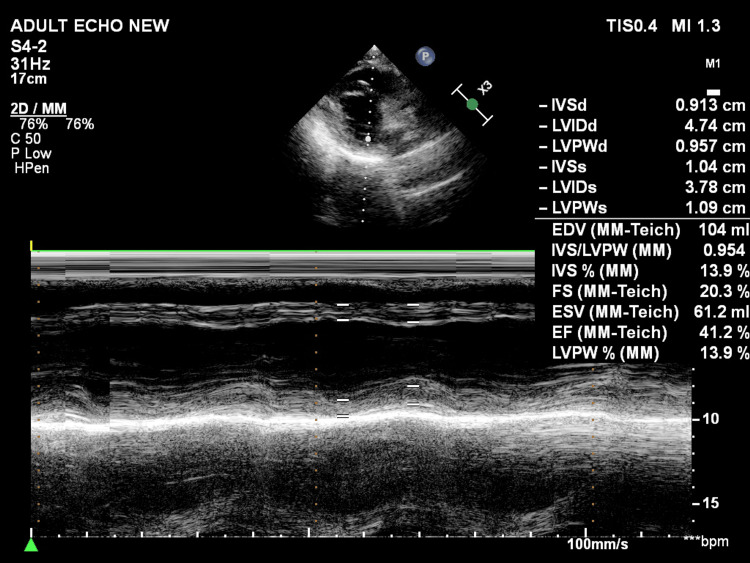
Repeat echocardiography (M-mode) 48 hours after revascularization showing improved LV systolic function with an EF of 40%-45% IVSd: interventricular septum thickness at end-diastole; LVIDd: left ventricular internal diastolic diameter; LVPWd: left ventricular posterior wall thickness in diastole; IVSd: interventricular septum thickness at end-systole; LVIDs: left ventricular internal systolic diameter; LVPWd: left ventricular posterior wall thickness in systole; EDV: end-diastolic volume; IVS: interventricular septum; LVPW: left ventricular posterior wall; FS: fractional shortening; ESV: end-systolic volume; EF: ejection fraction; LV: left ventricular

Repeat echocardiography (Figure 4) after 48 hours showed improved global LV systolic function with an EF of 40%-45%. The patient remained stable during hospitalization and was discharged after two days on dual antiplatelet therapy, high-intensity statins, beta-blockers, and optimized heart failure medications. At the four-week follow-up, he remained asymptomatic and had preserved functional capacity. The patient was subsequently referred for CABG after doing a positron-emission tomography scan for myocardial viability testing, which revealed hibernating but viable myocardium.

## Discussion

The myocardium receives blood supply predominantly from three coronary arteries: the LAD, RCA, and LCX arteries. Acute occlusion of any major artery leads to ischemic injury, progressing to infarction if prolonged [7]. Electrocardiographic (ECG) patterns aid in localizing the affected territory; ST-segment elevation in anterior leads typically signifies LAD involvement, whereas inferior lead changes suggest RCA or LCX pathology [8,9].

Simultaneous ischemic changes in multiple territories, as observed in our patient, are uncommon and may reflect complex coronary anatomy, prior infarct-related remodeling, or collateral circulation. In this case, the patient’s prior IWMI and subsequent chronic total occlusion (CTO) of the RCA left the inferior myocardial territory dependent on retrograde collateral perfusion from the LAD. Therefore, the acute LAD occlusion compromised blood flow to both anterior and inferior myocardial regions, manifesting with combined ECG changes.

Such presentations pose a diagnostic challenge, requiring urgent coronary angiography to differentiate between multivessel acute occlusions and a single culprit lesion supplying multiple territories [10]. Early identification and prompt revascularization are vital to salvaging viable myocardium and preventing extensive infarction. Previous reports have described similar cases where CTOs remained clinically silent until an acute event disrupted collateral flow, unmasking ischemia in multiple territories [11,12]. Smith et al. and Fiol et al. highlighted the diagnostic complexity and the need for modified ECG criteria or additional imaging to avoid misinterpretation.

Our case underscores the clinical significance of recognizing atypical ECG presentations in patients with prior coronary artery disease or interventions. It also emphasizes the role of collateral circulation in modifying both ischemic patterns and clinical outcomes. Comprehensive evaluation through angiography remains crucial, not only to confirm the culprit lesion but also to identify underlying chronic occlusions that may alter acute management strategies. Awareness of these mechanisms can guide clinicians toward timely and accurate interventions, ultimately improving patient prognosis.

## Conclusions

In summary, this case illustrates the diagnostic and management challenges posed by simultaneous ischemic changes in two coronary territories resulting from a single acute occlusion in the setting of preexisting CTO, mimicking multivessel ST-segment elevation myocardial infarction. It highlights the importance of recognizing atypical ECG findings that may arise due to collateral dependence, especially in patients with a history of prior MI or coronary interventions. Prompt coronary angiography and early revascularization were critical to achieving a favorable outcome in this patient. Clinicians must maintain a high index of suspicion for such presentations and understand the impact of chronic coronary pathology on acute ischemic events. A comprehensive diagnostic approach, integrating ECG interpretation, imaging, and angiographic evaluation, is essential to optimize patient care in complex MI scenarios.
